# Discovery of MurA Inhibitors as Novel Antimicrobials through an Integrated Computational and Experimental Approach

**DOI:** 10.3390/antibiotics11040528

**Published:** 2022-04-14

**Authors:** Fangyuan Zhang, Joshua Graham, Tianhua Zhai, Yanhong Liu, Zuyi Huang

**Affiliations:** 1 Department of Chemical and Biological Engineering, Villanova University, Villanova, PA 19085, USA; fzhang@villanova.edu (F.Z.); jgraha26@villanova.edu (J.G.); tzhai01@villanova.edu (T.Z.); 2 Molecular Characterization of Foodborne Pathogens Research Unit, Eastern Regional Research Center, U.S. Department of Agriculture, Wyndmoor, PA 19038, USA

**Keywords:** MurA inhibitors, antibiotic resistance, fosfomycin, *Listeria innocua*, *Escherichia coli*

## Abstract

The bacterial cell wall is essential for protecting bacteria from the surrounding environment and maintaining the integrity of bacteria cells. The MurA enzyme, which is an essential enzyme involved in bacterial cell wall synthesis, could be a good drug target for antibiotics. Although fosfomycin is used clinically as a MurA inhibitor, resistance to this antibiotic is a concern. Here we used molecular docking-based virtual screening approaches to identify potential MurA inhibitors from 1.412 million compounds from three databases. Thirty-three top compounds from virtual screening were experimentally tested in *Listeria innocua* (Gram-positive bacterium) and *Escherichia coli* (Gram-negative bacterium). Compound 2-Amino-5-bromobenzimidazole (S17) showed growth inhibition effect in both *L. innocua* and *E. coli*, with the same Minimum Inhibitory Concentration (MIC) value of 0.5 mg/mL. Compound 2-[4-(dimethylamino)benzylidene]-*n*-nitrohydrazinecarboximidamide (C1) had growth inhibition effect only in *L. innocua*, with a MIC value of 0.5 mg/mL. Two FDA-approved drugs, albendazole (S4) and diflunisal (S8), had a growth inhibition effect only in *E. coli*, with a MIC value of 0.0625 mg/mL. The identified MurA inhibitors could be potential novel antibiotics. Furthermore, they could be potential fosfomycin substitutes for the fosfomycin-resistant strains.

## 1. Introduction

*Listeria monocytogenes* is a Gram-positive, foodborne pathogen [1]. The bacterium is prevalent in natural environments and a transitory resident of the intestinal tract [2]. Its ability to grow in low moisture, high salt concentrations, and refrigerated settings (−0.5 to 9.3 °C) poses a serious issue for the processed food industry, especially ready-to-eat (RTE) foods [3]. *L. monocytogenes* expresses internalin, a surface protein which interacts with E-cadherin in the intestine, brain, and fetoplacental barriers allowing passage through epithelial cells [4]. While *L. monocytogenes* is linked to a mild, febrile illness, immunocompromised hosts, such as kids, pregnant women, and elderly people commonly succumb to listeriosis or a much more serious illness such as sepsis, meningitis, or encephalitis. These illnesses can lead to hospitalizations and account for fatal foodborne outbreaks [1,5,6,7]. Unfortunately, the fact that food-derived *L. monocytogenes* strains are adapted to antibiotic treatments poses another complication in the treatment of listeriosis. The persistence of the bacterium makes it an important target for research and intervention.

Peptidoglycan is a component of the bacterial cell wall responsible for mechanical strength and resistance to environmental stress [8]. The fact that peptidoglycan biosynthesis is necessary for bacterial growth and is well conserved across bacterial species makes it a common antibiotic target [8]. Since the enzyme MurA catalyzes the first step in peptidoglycan synthesis, MurA becomes a key target to inhibit bacterial replication [9]. MurA is specifically responsible for transferring enolpyruvate from phosphenol pyruvate (PEP) to UDP-*n*-acetyl glucosamine (UNAG) that catalyzes the conversion to UDP-*n*-Acetyl muramic acid [9]. A handful of inhibitors have successfully inhibited the MurA enzyme, and the most well-known MurA inhibitor is fosfomycin [9]. In particular, fosfomycin is a well-known therapeutic antibiotic for treatment against listeriosis [9]. Interaction of fosfomycin with the MurA enzyme found in *L. monocytogenes* results in a covalent bond with the active cysteine-115 side chain, and the subsequent inhibition of the peptidoglycan biosynthesis causes cell death [10]. While fosfomycin has been a successful antibiotic against *L. monocytogenes* in the past, it now faces issues with antibiotic resistance, difficulties navigating the multi-protein structure of the Mur enzyme, or a lack of specificity [9]. FosX is one of the major genes causing these issues. It catalyzes the hydration of fosfomycin and makes it unable to function as an antibiotic [11]. FosX is found in many bacteria including *L. monocytogenes* and devalues the fosfomycin treatment. There are 31 known mutations in the *FosX* gene which makes it difficult to intercept and inhibit the bacterial growth; therefore, there is a need to discover different pathways to limit the growth of *L. monocytogenes* [11]. This study aims to find effective inhibitors of the MurA enzyme that can be used as new antibiotics.

Since millions of molecules exist in chemical compound libraries [12,13], it is costly and time-consuming to evaluate each compound for its inhibition against the MurA protein experimentally. Automated molecular docking provides a solution for this, as it offers a quick computational evaluation of the binding affinity between small-molecule ligands and the MurA protein with a known three-dimensional crystalline structure. Recent optimization of algorithms and scoring functions have permitted more reliable assessments [14]. Previous studies and tests indicated that Molsoft ICM performs outstandingly for covalent docking, docking pose, and energy prediction [14]. On the basis of the ICM-based docking program [15,16], the MurA enzyme was in-silico docked by compounds from three databases in this work, including FDA-approved drugs, Sigma, and ChemBridge, to identify a list of MurA inhibitors that could be naturally and economically sourced.

Since the MurA enzyme plays a significant role in the cell wall synthesis of both Gram-positive and Gram-negative bacteria, inhibitors of MurA enzyme may become novel antibiotics that inhibit the growth of a broad-spectrum of bacteria. This is implied by the conservation of the structure of the MurA structure across a variety of bacterial strains [17]. The analysis of X-ray crystal structures of MurA in a plethora of species determined that Arg120 and three glycine residues are conserved in the Cys115 loop where fosfomycin is known to inhibit the enzyme [17]. In addition, most residues at the interdomain cleft are highly conserved [17]. *L. innocua*, which is genetically, morphologically, and biochemically similar to *L. monocytogenes* (Appendix A, Figure A2a) [18,19,20], was selected to evaluate the inhibition effect of the identified compounds against Gram-positive bacteria. To evaluate the impact of similarity between MurA proteins across species on compound inhibition effect, *Escherichia* *coli* K12 was selected as the representative for Gram-negative bacterium for being tested against the identified inhibitors. The compounds identified in this work may serve as good candidates for inhibiting *L. monocytogenes* and other pathogens.

## 2. Results

### 2.1. Inhibitors Identified from the Ligand-Protein Docking Computation

**Figure A1:**
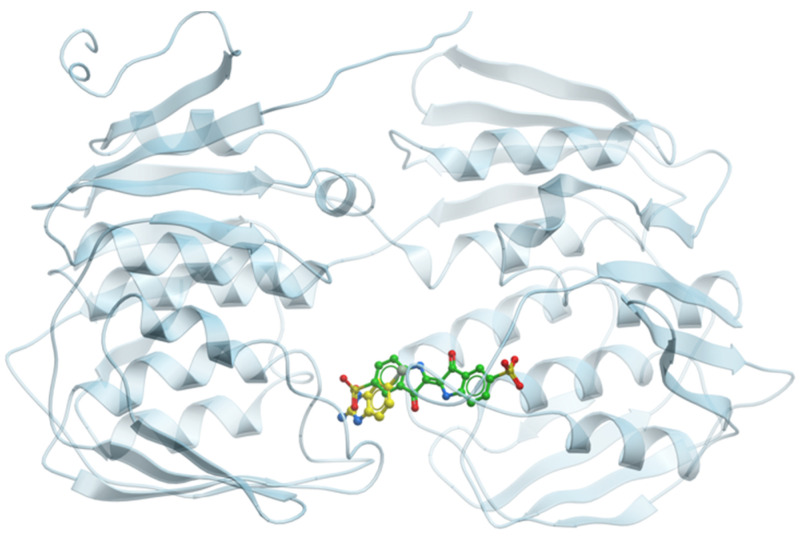
A putative MurA inhibitor S17 binds MurA substrate-binding site. The compound S17 (yellow) and original substrate of MurA, UNAG (Green), was docked into the MurA enzyme (sky blue).

The 1.412 million compounds from the three databases (i.e., FDA-approved drugs, Sigma database, and ChemBridge database) were docked into the MurA protein and evaluated for their binding affinities. UNAG, a natural substrate of the MurA enzyme, had a docking score of −27.77 kcal/mol. In total, 2189 compounds gave docking scores lower than –32kcal/mol, which indicated a stronger binding affinity than UNAG. As shown in Figure A1, the binding site of one selected compound resembled that of UNAG, which indicated that this selected compound could be a potential competitive inhibitor of UNAG for MurA. The compounds were further selected and validated by experiments.

**Figure 1:**
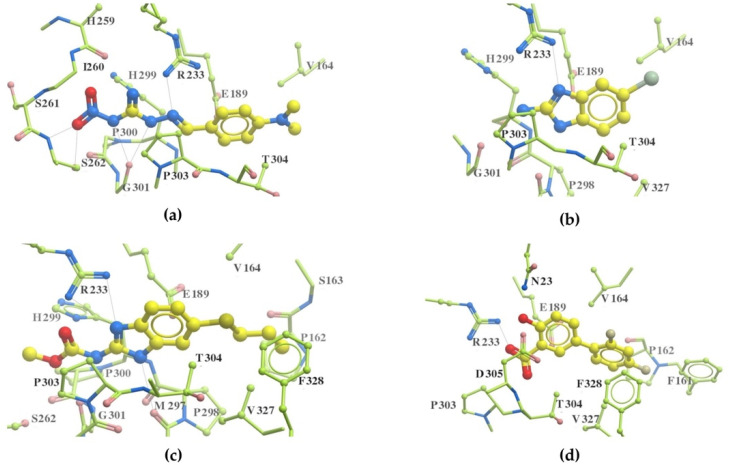
Putative MurA inhibitors bind to MurA substrate-binding site. The compounds (yellow) were docked into the UNAG-binding site of MurA enyzme (green) and evaluated binding affinity. Based on docking models, (**a**) compound C1, 2-[4-(dimethylamino)benzylidene]-*N*-nitrohydrazinecarboximidamide, binds to residues R233, S261, and G301; (**b**) compound S17, 2-amino-5-bromobenzimidazole, interacts with residues R233 through hydrogen bond; (**c**) compound S4, albendazole, has hydrogen bonding with residues R233 and M297; (**d**) compound S8, diflunisal, forms hydrogen bonds with residues R233. Hydrogen bonds are marked as a black dotted line.

Figure 1 illustrates the docked conformation of four inhibitors of MurA and ligand–protein interactions at the atomic level. The four inhibitors were predicted to interact with Arg233 through hydrogen bonds, which was considered to be a conserved ligand–protein interaction. Interestingly, these four inhibitors had polar functional groups attached to aromatic rings. The polar groups might act as both H-bond donors and acceptors, interacting with polar residues, e.g., arginine or serine. The aromatic rings help interact with nonpolar residues such as valine and phenylalanine. The intermolecular interactions might enhance the binding affinity, which helps identified compounds competitively bind to UNAG-binding site and inhibit MurA activity.

### 2.2. Growth Inhibition Assay Using L. innocua

**Figure 2:**
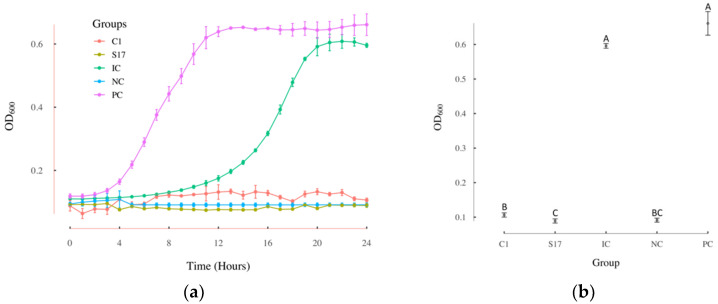
Compounds effective in growth inhibition assay using *L. innocua*. (**a**) The growth curves of *L. innocua* over 24 h under different treatment conditions; (**b**) the OD_600_ values of *L. innocua* at 24 h under different treatment conditions. ANOVA revealed significant differences among the different groups (F_4,10_ = 972.8, *p* = 6.63 × 10^−13^). The results of the Kruskal–Wallis test are represented with letters (different capital letters = significantly different groups); PC: positive control group; NC: negative control group; IC: the known inhibitor fosfomycin 0.5 mg/mL group; C1: 2-[4-(dimethylamino) benzylidene]-*N*-nitrohydrazinecarboximidamide 0.5 mg/mL group; S17: 2-amino-5-bromobenzimidazole 0.5 mg/mL group; OD_600_: the absorbance at 600 nm; *n* = 3; error bars represent standard deviations.

Due to the available resources, the top 33 compounds of the 2189 compounds identified from the computational platform were further evaluated in the growth inhibition test in *L. innocua*. The detailed information of these 33 compounds can be found in an Appendix A, Table A1. As shown in Figure 2a, two of the tested compounds showed apparent inhibitions of *L.* *innocua* growth. Figure 2b presents the results of the ANOVA test and the Kruskal–Wallis test of the OD_600_ values of each group at 24 h. The *p* value of the ANOVA test was 6.63 × 10^−13^, which indicated that at least two groups among the C1, S17, IC, NC, and PC groups were significantly different from each other. Since the data were not normally distributed, the nonparametric Kruskal–Wallis test was conducted. A Chi squared value of 12.86 and a *p* value of 0.012 were returned in this test. As shown in Figure 2b, the known inhibitor control group (IC) treated with 0.5 mg/mL fosfomycin did not show significant difference from the positive control groups. Compounds C1 and S17 groups showed significant differences from both the positive control group and IC groups, proving significant inhibition of the growth of *L. innocua*, and significantly better inhibition effects than the known inhibitor, fosfomycin. The growth reduction rate of fosfomycin was 10.45% in *L.* *innocua*, while the growth reduction rate of 2-amino-5-bromobenzimidazole (S17) was 100% and the growth reduction rate of 2-[4-(dimethylamino)benzylidene]-*N*-nitrohydrazinecarboximidamide (C1) was 96.62%.

### 2.3. Minimum Inhibitory Concentration (MIC) Assay Using L. innocua

**Figure 3:**
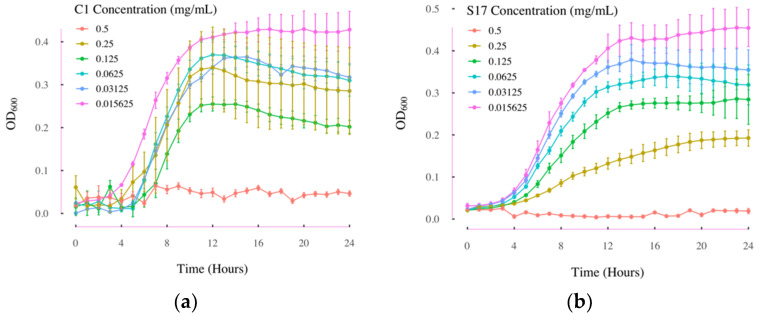
The growth curves of L. innocua over 24 h in MIC test. (**a**) Growth curves of L. innocua under different concentration of compound C1 treatment over 24 h; (**b**) growth curves of L. innocua under different concentration of compound S17 treatment over 24 h. C1: 2-[4-(dimethylamino)benzylidene]-N-nitrohydrazinecarboximidamide; S17: 2-Amino-5-bromobenzimidazole; OD_600_: the absorbance at 600 nm; *n* = 3; Error bars represent standard deviations.

The two compounds showing apparent growth inhibition effects were further tested for their MIC. In Figure 3a, *L. innocua* did not show obvious growth over 24 h when cultured together with compound C1 at the concentration of 0.5 mg/mL. However, the *L. innocua* had obvious growth from 5 h to 10 h during the 24-h culture under the C1 treatment at concentration of 0.25 mg/mL. Thus, the MIC of C1 on *L. innocua* was around 0.5 mg/mL. Similarly, as shown in Figure 3b, *L. innocua* did not have obvious growth only when treated with 0.5 mg/mL of compound S17, which indicated that the MIC of S17 on *L. innocua* was around 0.5 mg/mL.

### 2.4. Growth Inhibition Assay Using E. coli

**Figure 4:**
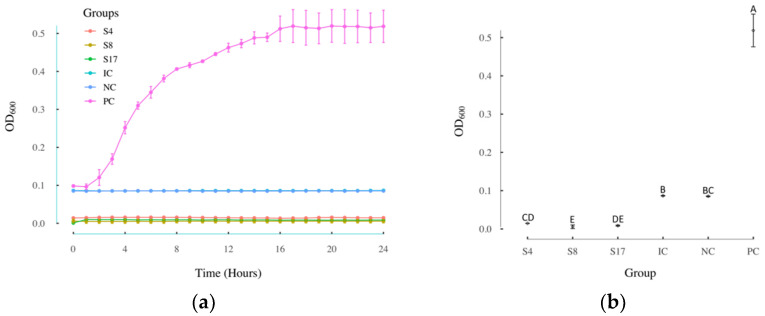
Compounds effective in growth inhibition assay using *E. coli*. (a) The growth curves of *E. coli* over 24 h under different treatment conditions; (b) the OD_600_ values of *E. coli* at 24 h under different treatment conditions. ANOVA revealed significant differences among the different groups (F_5,12_ = 385.3, *p* = 8.19 × 10^−13^). The results of the Kruskal–Wallis test are represented with letters (different capital letters = significantly different groups); PC: positive control groups; NC: negative control groups; IC: the known inhibitor fosfomycin 0.5 mg/mL groups; S4: albendazole 0.5 mg/mL groups; S8: diflunisal 0.5 mg/mL groups; S17: 2-amino-5-bromobenzimidazole 0.5 mg/mL groups; OD_600_: the absorbance at 600nm; *n* = 3; error bars represent standard deviations.

The 33 compounds tested in *L. innocua* were also tested in *E. coli* for their growth inhibition effect in Gram-negative bacterium. As shown in Figure 4a, three compounds showed apparent growth inhibition in *E. coli*. Figure 4b presents the results of an ANOVA test and a Kruskal–Wallis test on the OD_600_ values of each group at 24 h. The *p* value from the ANOVA test was 8.19 × 10^−13^, which indicates that at least two groups among the S4, S8, S17, IC, NC, and PC groups were significantly different from each other. Since the data were not normally distributed, the nonparametric Kruskal–Wallis test was used, which yielded a Chi square of 16.13 and *p* value of 0.0065. The different significance levels are represented by capital letters in Figure 4b. All three of the selected compounds S4, S8, and S17 groups showed significant difference with the positive group. The growth inhibition rates of 2-amino-5-bromobenzimidazole (S17), diflunisal (S8), and albendazole (S4) were 100% at the concentration of 0.5 mg/mL.

### 2.5. MIC Assay Using E. coli

**Figure 5:**
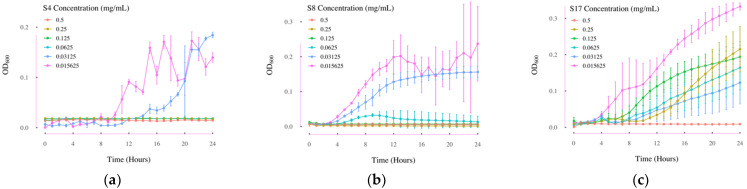
The growth curves of *E. coli* over 24 h in MIC test. (**a**) Growth curves of *E. coli* under different concentrations of compound C4 treatment over 24 h; (**b**) growth curves of *E. coli* under different concentrations of compound S8 treatment over 24 h; (**c**) growth curves of *E. coli* under different concentrations of compound S17 treatment over 24 h. S4: albendazole; S8: diflunisal; S17: 2-amino-5-bromobenzimidazole; OD_600_: the absorbance at 600nm; *n* = 3. Error bars represent standard deviations.

The MIC value of the three compounds showing apparent growth inhibition effects in *E. coli* were further tested using an MIC assay. As shown in Figure 5a, *E. coli* had no obvious growth when treated with 0.0625 mg/mL or higher concentrations of S4. However, when the concentration of S4 compound was decreased to 0.03125 mg/mL, *E. coli* started to grow after 13 h of culture. Therefore, the MIC of S4 should be 0.0625 mg/mL. Similarly, as shown in Figure 5b, *E. coli* had no obvious growth when treated with 0.0624 mg/mL or higher concentration of S8, which indicates that the MIC of compound S8 should also be 0.0625 mg/mL. As shown in Figure 5c, when *E. coli* was treated with 0.25mg/mL of compound S17, *E. coli* had growth over the 24 h, but not under the treatment of 0.5mg/mL compound S17 treatment. Therefore, the MIC value of compound S17 in *E. coli* should be around 0.5 mg/mL.

## 3. Discussion

In this study, we conducted a molecular docking-based virtual screening to narrow down the potential hit compounds and verified their growth inhibition effects experimentally. This integration method highly increased the compound-screening efficiency and broadened the range of compounds that could be screened. Most notably, to our knowledge, this is the first study to identify the growth inhibition effect of the two compounds, 2-amino-5-bromobenzimidazole and 2-[4-(dimethylamino)benzylidene]-N-nitrohydrazinecarboximidamide. Our study provides compelling evidence that these two compounds might be new potential antimicrobials and worth further investigation. This study also identified the possible novel function as antibiotics for the two FDA approved drugs, albendazole and diflunisal.

As shown in Table 1, two MurA inhibitors, 2-[4-(dimethylamino)benzylidene]-*N*-nitrohydrazinecarboximidamide and 2-amino-5-bromobenzimidazole, obviously inhibited the growth of the Gram-positive bacterium *L. innocua.* In literature, there is no toxicity or experimental data on 2-Amino-5-bromobenzimidazole. However, 2-aminobenzimidazole derivatives are recognized for immunotropic, diuretic, antihistamine, and antiviral characteristics [21]. The MSDS data from vendors are unavailable [22]. Similarly, no scientific evidence of prior 2-[4-(dimethylamino)benzylidene]-*N*-nitrohydrazinecarboximidamide usage has been identified.

In addition to 2-[4-(dimethylamino)benzylidene]-*N*-nitrohydrazinecarboximidamide, two more MurA inhibitors were found in this study with a strong inhibition effect on the growth of the Gram-negative bacterium *E. coli*. Among them are albendazole and diflunisal, both of which are FDA-approved drugs. Albendazole is an anthelmintic drug with potential cytocidal properties [23,24]. It is known for the treatment of echinococcosis, hydatid cyst, and neurocysticercosis via its metabolism to albendazole sulphoxide in the human body [23,25,26]. Albendazole has an affinity for rapidly dividing cells, and this causes concerns over toxicity to bone marrow and the intestinal epithelium [27]. The FDA also recorded rare fatalities from granulocytopenia or pancytopenia and issues with aplastic anemia and agranulocytosis, indicating a need for close monitoring of patient blood counts [24]. Studies on albendazole’s effects on cystic echinococcosis have also suggested that liver function and hair may be affected, but bone marrow was the biggest safety concern [26]. Animal trials conducted on mice, rats, hamsters, and rabbits resulted in mortality with doses ranging from 500 to 10,000 mg/kg, indicating species-dependent adverse effects [24]. Diflunisal is a salicylic acid derivative known for its analgesic, anti-inflammatory, and uricosuric activity [28,29]. It inhibits the second phase of platelet aggregation from adenosine diphosphate, and it is commonly used as a pain killer [29,30]. Clinical studies on diflunisal suggest gastrointestinal (GI), central nervous system (CNS), hypertension, and edema effects, but overall, diflunisal is tolerated as well as aspirin and other pain killers [28,30]. Diflunisal has been shown to stabilize transthyretin and play a role in amyloidogenesis [31]. It is fatal if diflunisal is mixed with large doses of aspirin; however, studies with the diflunisal dose of 8 mg/kg/day in beagle puppies and 140 mg/kg/day in rats showed low mortality rates [30].

Toxicity of the four inhibitors was evaluated through an in silico approach in ICM. A program named Toxscore calculates potential toxicity based on substructure and indicates toxic functional groups [32]. Compound S17, S4, and S8 indicated no or less toxicity as the Toxscores were less than 1. Nonetheless, inhibitor C1 was detected to have toxic functional groups including nitro, imines, and hydrazone. Therefore, in vivo toxicity tests should be conducted for validation.

**Figure A2:**
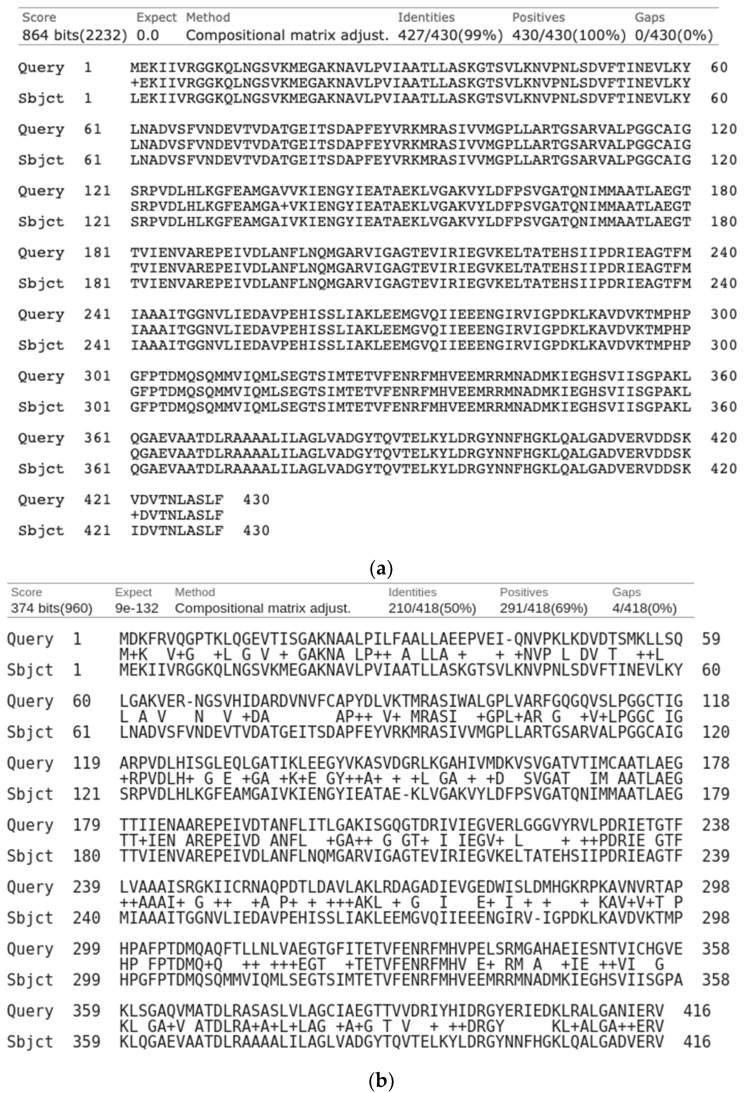
MurA protein sequence comparisons: (**a**) Comparison of protein sequence of MurA in *Listeria monocytogenes* (Q8Y4C4) and *Listeria innocua* (Q927W7) by using BlastP. Query = protein sequence of MurA in *Listeria monocytogenes*; Sbjct = protein sequence of MurA in *Listeria innocua*. 99% sequence identity; (**b**) comparison of protein sequence of MurA in *E coli* (P0A749) and *L. innocua* (Q927W7) by using BlastP. Query = protein sequence of MurA in *E coli*; Sbjct = protein sequence of MurA in *Listeria innocua*. 50% sequence identity.

The sequences of MurA protein between *E. coli* and *L. innocua* were 50% identical, as shown in Figure A2b. By comparison of MurA protein structure from *E. coli* and *L. innocua*, particularly the residues around UNAG-binding site, it was found that four residues were variant, namely, W95*^E.coli^*-V97*^L.innocua^*, A119*^E.coli^*-S121*^L.innocua^*, K160*^E.coli^*-F161*^L.innocua^*, and V161*^E.coli^*-P162*^L.innocua^*, which are marked in Figure A2a. In addition, three same residues indicated different conformations including R120*^E.coli^*-R122*^L.innocua^*, R91*^E.coli^*-R90*^L.innocua^*, and K22*^E.coli^*-K22*^L.innocua^*. The variation and rotation of the residues around the UNAG-binding site would impact inhibitors binding, which could be one reason that compound C1, S17, and S4 had different inhibitory effects in *E. coli* and *L. innocua*

As shown in Table 1, although compound S17 was not the compound with the lowest MIC, especially in *E. coli*, it was able to inhibit the growth of both Gram-positive and Gram-negative bacteria. This might be due to its small size when compared to other compounds. The small size enables its diffusion across the cell wall and cell membrane(s) of bacteria. In addition, it was also noticed that the growth inhibition effect of S17 was better in Gram-negative *E. coli* than in the Gram-positive bacterium *L. innocua* under the same treatment conditions. Based on previous research, the presence of two active MurA forms in Gram-positive bacteria results in higher MurA expression levels [33]. Accordingly, higher concentration of a competitive inhibitor might be needed to achieve similar inhibition effects. In addition, the MurA enzyme from Gram-negative bacteria, especially *E. coli*, was proven to be more efficient than the MurA enzyme from Gram-positive bacteria [34,35].

In future studies, two main parts of research could be considered. Further research validating the growth inhibition effects of the four identified inhibitors with other bacterial strains could further prove the application value of these inhibitors. Compound S17 could be tested with more bacterial strains, both Gram-positive and Gram-negative bacteria, to further verify our hypothesis that this compound might be a broad-spectrum antibiotic. For the remaining compounds, compound C1 could be tested using other gram-positive bacteria, such as *L. monocytogenes*, and *Streptococcus pyogenes*. Compound S4 and S8 could be tested in other Gram-negative bacteria, such as *Salmonella enterica*. If our identified compounds could also have growth inhibition effects in the foodborne pathogens, those compounds could be potential antibiotics for foodborne disease control and relieve the problems of antibiotic resistance. In addition, future research on identification the toxicity and pharmaceutical effects of 2-amino-5-bromobenzimidazole and 2-[4-(dimethylamino)benzylidene]-*N*-nitrohydrazinecarboximidamide is recommended based on the results of this study.

## 4. Materials and Methods

### 4.1. Identification of MurA Inhibitors through Molecular Docking Based Virtual Screening

**Figure A3:**
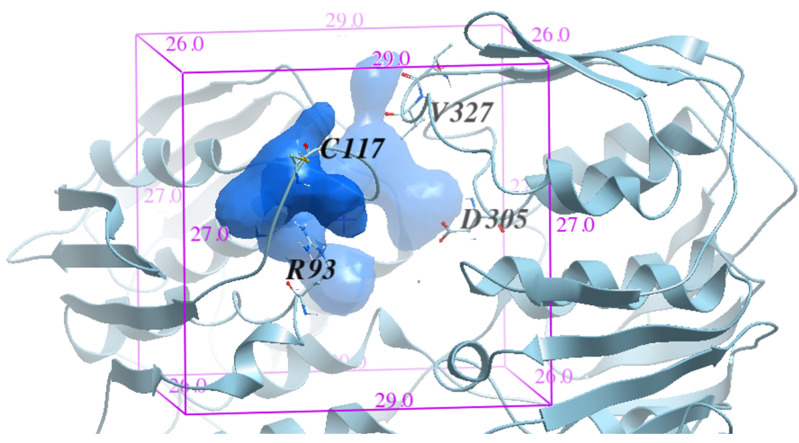
The docking box generated for docking-based virtual screening. The ligand binding pocket (blue) was predicted by ICM with a recommended tolerance level 4.6, which covers the enzyme active site (C117, R93, D305, and V327). The docking box (purple) was generated around the pocket with a size of 29 × 26 × 27 Å. The initial docking position was at the center of the box.

In the virtual screening, the MurA gene in *L. monocytogenes* EGD-e strain was obtained from Uniplot with the ID of Q8Y4C4. The model for virtual screening was the protein structure of MurA in *L. monocytogenes* serovar 1/2a (strain ATCC BAA-679 / EGD-e), which was found in Protein Data Bank with the ID 3R38. The protein receptor was modified based on the structure 3R38 by removing sulfate ion, deleting water, and adding hydrogens. The following residues were further optimized: three protonation states and two rotations of all histidine (His) residues and 180-degree flip of asparagine (Asn) and glutamine (Gln) residues were implemented to minimize the global energy. Particularly, both His41 and His163 at the active site were in Nδ1-protonated π tautomer state. The ligand binding pocket was predicted by icmPocketFinder with a recommended tolerance level of 4.6 by ICM. As shown in Appendix A, Figure A3, the pocket covering enzyme active site C117, R93, D305, and V327 was selected. The docking box was generated with a size of 29 × 26 × 27 Å and the initial docking position was placed at the center of the box, shown in Appendix A, Figure A3. Natural substrate UNAG was docked into the receptor and got docking score –28.77 kcal/mol. The docking pose gave an RMSD of 0.18 Å relative to ligand conformation of UNAG in structure 3KR6. Docking software Molsoft ICM-Pro 3.7b (Molsoft, San Diego, CA, USA) was used to conduct the virtual screening on the same protocol as previous published research [15]. Three databases, FDA-approved drugs (2000 compounds), Sigma (10,000 compounds), and ChemBridge (1.4 million compounds), were used as the inhibitor candidates in the in silico screening. The FDA-approved drugs are well-studied in terms of efficacy and safety. Repurposing existing drug is an efficient strategy to explore advanced uses. Sigma–Aldrich provides best-in-class chemical drugs for experiments and Chembridge Corporation has over 1.4 million diverse and target-focused screening compounds for small molecule drug discovery. Therefore, the FDA-approved drugs database was set as first trial in virtual drug screening followed by commercially available compound libraries. Compounds were first filtered by “Lipinski’s rules of five” and around 1 million compounds were maintained and docked into the protein receptor. The virtual screening was conducted with common ICM settings including scoring function 2005 and docking effort 1. The compounds with good docking scores lower than −32 kcal/mol were retained (as recommended by ICM), which might have a higher binding affinity than ligand UNAG (−27.77 kcal/mol).

### 4.2. Bacterial Strains and Culture Conditions

*L. innocua* strain used in this study was purchased from American Type Culture Collection (ATCC, Manassas, VA, USA). At the start of each experiment, a single colony of *L. innocua* was cultured overnight in 3 mL brain heart infusion broth (BHI, Sigma–Aldrich Inc., St. Louis, MO, USA) at 37 °C with 200 rpm agitation. *E. coli* wild type K12 strain used in this study was purchased from ATCC. At the start of each experiment, a single colony of *E. coli* was cultured overnight in 3 mL Lysogeny broth (LB, Sigma–Aldrich Inc., St. Louis, MO, USA) at 37 °C with 200 rpm agitation.

### 4.3. Chemical Stock Solution Preparation and Storage

The tested compounds, as shown in Appendix A, Table A1, were purchased from ChemBridge Corporation (San Diego, CA, USA) or Sigma–Aldrich (St. Louis, MO, USA). Each compound to be tested was dissolved in sterilized dimethyl sulfoxide (DMSO, Sigma–Aldrich Inc., St. Louis, MO, USA) or sterilized water, as recorded in Table A1, to the final concentration of 10 mg/mL and stored at 4 °C until further use. Whether the compound was dissolved in DMSO or water was decided based on the water solubility of each compound.

### 4.4. Bacterial Growth Inhibition Study

A single colony of *L. innocua* was used to be cultured in 3 mL BHI overnight and diluted 1000-fold for the bacterial cell growth inhibition study. Then, 10 μL of 10 mg/mL a compound stock solution was added into one well in 96-well plate together with 190 μL of 1000-fold diluted *L. innocua* overnight culture as the experimental groups. The negative control groups were set up by adding 10 μL sterilized DMSO and 190 μL BHI into each well of negative control groups in the 96-well plate. The positive control groups were set up by adding 10 μL sterilized DMSO and 190 μL of 1000-fold diluted *L. innocua* overnight culture into each well of negative control groups in the 96-well plate. The background color group was set up by adding 10 μL of 10 mg/mL compound stock solution into one well in 96-well plate together with 190 μL of distilled water. The 96-well plate holding the experimental groups, the negative control groups, the positive control groups, and the background color groups was placed into microplate reader (BioTek Instruments, Inc., Winooski, VT, USA) to record the OD_600_ value every hour for 24 h under 37 °C with linear agitation for 5 s before each read. The microplate reader used the software Gen5 (version 3.00.19, BioTek Instruments, Inc., Winooski, VT, USA) for data recording. All of the selected compounds were screened with only 1 group in the cell growth inhibition screening. The groups with obvious reduction of bacteria growth were selected to repeat the growth inhibition test in triplicate. Considering the background color of some tested compounds, the OD_600_ value of inhibitor group was subtracted from the OD_600_ value of the background color group from original OD_600_ value at the same time point. The OD_600_ values of each group at 24 h were used to conduct the ANOVA to test the differences among groups. For the data that indicated at least two groups were significantly different by ANOVA, the Kruskal–Wallis test was conducted to test the differences among the groups. The ANOVA and Kruskal–Wallis tests were conducted and plotted by using R (version 4.1.1). The growth inhibition effect was analyzed by calculating the growth reduction rate (i.e., *r*) based on a published method, as in Equation (1) [16].

(1) $$r=\frac{\left[\Delta OD_{600}\left(\mathrm{PC}\right)-\Delta OD_{600}\left(\mathrm{Inhibitor}\;\mathrm{group}\right)\right]}{\Delta OD_{600}\left(\mathrm{PC}\right)}\times 100\%$$

where PC stands for the positive control group. The ΔOD_600_ was calculated by subtracting the OD_600_ value recorded at the time 0 from the OD_600_ value recorded at the 24 h.

### 4.5. Minimum Inhibitory Concentration (MIC) Assays

The stock solution of the chemicals to be tested was diluted using 2-fold serial dilution method [36]. First, 10 μL stock solution/diluted stock solution was added into one well in 96-well plate with 190 μL of 1000-fold diluted *L. innocua* or *E. coli* overnight culture. Negative control groups and positive groups were the same setup as mentioned in Section 4.4. The plate carrying the experimental groups and control groups was incubated in the microplate reader to record the OD_600_ value every hour for 24 h under 37 °C with linear agitation for 5 s before each read. Each group was repeated three times. The MIC data collected by Gen5 were analyzed and plotted using R (version 4.1.1).

## 5. Conclusions

To discover new inhibitors of MurA to inhibit the growth of bacteria, a computational virtual screening was implemented, followed by experimental verification to test the bacteria growth inhibition effects. There were 1.412 million compounds screened in the computational virtual screening, among which 2189 compounds were identified. The top 33 identified compounds from computation were further evaluated by experimentation, and four inhibitors were identified. Among the four inhibitors, 2-amino-5-bromobenzimidazole is the only inhibitor that worked on both a Gram-positive bacterium (i.e., *L. innocua*) and a Gram-negative bacterium (i.e., *E. coli*) with a MIC of 0.5 mg/mL for both strains. 2-[4-(dimethylamino)benzylidene]-*N*-nitrohydrazinecarboximidamide showed growth inhibition in *L. innocua*, with a MIC of 0.5 mg/mL. The FDA-approved drugs albendazole and diflunisal showed growth inhibition in *E. coli*, with a MIC of 0.0625mg/mL. In future studies, the growth inhibition effects could be tested using more bacteria strains, especially pathogenic strains to further verify the possible use of those identified inhibitors as antibiotics. Furthermore, the toxicity test and preclinical tests of those chemicals should also be conducted to verify the safety and efficacy of those inhibitors.

## Figures and Tables

**Table 1:** Summary of the bacterial growth inhibition effects of identified MurA inhibitors.

Structure	Compound Number in this Study	Name of Compound	Growth Inhibition in *L. innocua*		Growth Inhibition in *E. coli*	
			Effective	MIC (mg/mL)	Effective	MIC (mg/mL)
* 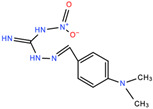 *	C1	2-[4-(dimethylamino)benzylidene]-N-nitrohydrazinecarboximidamide	Yes	0.5	No	-
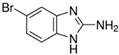	S17	2-Amino-5-bromobenzimidazole	Yes	0.5	Yes	0.5
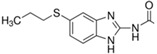	S4	Albendazole	No	-	Yes	0.0625
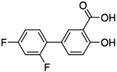	S8	Diflunisal	No	-	Yes	0.0625

## Data Availability

The original contributions presented in the study are included in the article/Appendix A, further inquiries can be directed to the corresponding authors.

## Appendix A

**Table A1:** Information of selected compounds to be experimentally tested.

Compound Number in this Study	Compound Name	Database	Vender	Solvent Used
S1	Mebendazole	FDA-approved	Sigma	DMSO
S2	Indigo carmine	FDA-approved	Sigma	H_2_O
S3	Olsalazine sodium	FDA-approved	Sigma	H_2_O
S4	Albendazole	FDA-approved	Sigma	DMSO
S5	Iobenguane sulfate	FDA-approved	Sigma	H_2_O
S6	Balsalazide disodium salt hydrate	FDA-approved	Sigma	H_2_O
S7	Lodoxamide	FDA-approved	Sigma	DMSO
S8	Diflunisal	FDA-approved	Sigma	DMSO
S9	TRIPHENYLPHOSPHINE-3,3’,3’’-TRISULFONIC acid trisodium salt (TPPTS)	Sigma	Sigma	H_2_O
S10	3-(4-(Benzyloxy)phenyl)-1H-pyrazole-5-carbohydrazide	Sigma	Sigma	DMSO
S11	3-(2-Pyridyl)-5,6-diphenyl-1,2,4-triazine-p,p′-disulfonic acid monosodium salt hydrate	Sigma	Sigma	H_2_O
S12 *	Fosfomycin disodium salt	Sigma	Sigma	H_2_O
S13	Tris(3,3′,3″-phosphinidynetris(benzenesulfonato) palladium(0) nonasodium salt nonahydrate	Sigma	Sigma	H_2_O
S14	Glutaraldehyde sodium bisulfite addition	Sigma	Sigma	DMSO
S15	[5-(2-Methyl-5-fluorophenyl)furan-2-ylcarbonyl]guanidine	Sigma	Sigma	DMSO
S16	4-Methyl-5-(sulfomethylamino)-2-(2-thiazolylazo)benzoic acid	Sigma	Sigma	H_2_O
S17	2-Amino-5-bromobenzimidazole	Sigma	Sigma	DMSO
S18	3-Hydroxy-2-(6-methylquinazolin-4-ylamino)propanoic acid hydrochloride	Sigma	Sigma	H_2_O
S19	2,3-Pyrazinedicarboxamide	Sigma	Sigma	DMSO
S20	(R)-(–)-2-Aminobutanamide hydrochloride	Sigma	Sigma	DMSO
C1	2-[4-(dimethylamino)benzylidene]-N-nitrohydrazinecarboximidamide	ChemBridge	ChemBridge	DMSO
C2	disodium 4,4’-(2-oxo-2,3-dihydro-1H-imidazole-4,5-diyl)dibenzenesulfonate	ChemBridge	ChemBridge	DMSO
C3	4-[2-(aminocarbonyl)carbonohydrazonoyl]-2-methoxyphenyl 3-chloro-1-benzothiophene-2-carboxylate	ChemBridge	ChemBridge	DMSO
C4	N-(2-amino-2-oxoethyl)-4-[(1-hydroxycyclohexyl)ethynyl]benzamide	ChemBridge	ChemBridge	DMSO
C5	3-(1,2-dihydro-5-acenaphthylenyl)-1H-pyrazole-5-carbohydrazide	ChemBridge	ChemBridge	DMSO
C6	2-[4-(6-bromo-4-phenyl-2-quinolinyl)phenoxy]acetohydrazide	ChemBridge	ChemBridge	DMSO
C7	4-({[5-(2-carboxyvinyl)-2,3-dimethoxyphenyl]sulfonyl}amino)benzoic acid	ChemBridge	ChemBridge	DMSO
C8	ethyl 2-{[4-(aminocarbonyl)phenyl]hydrazono}-3-oxobutanoate	ChemBridge	ChemBridge	DMSO
C9	4-({2-cyano-2-[4-(3-nitrophenyl)-1,3-thiazol-2-yl]vinyl}amino)benzamide	ChemBridge	ChemBridge	DMSO
C10	2-[(aminocarbonyl)amino]-N-{2-[4-(hydroxymethyl)piperidin-1-yl]-5,6,7,8-tetrahydroquinazolin-5-yl}acetamide	ChemBridge	ChemBridge	DMSO
C11	N-{2-[2-(4-chlorophenyl)-1,4,6,7-tetrahydro-5H-imidazo[4,5-c]pyridin-5-yl]-2-oxoethyl}urea	ChemBridge	ChemBridge	DMSO
C12	2-methoxy-3’-{[(1-methyl-2-oxopyrrolidin-3-yl)amino]carbonyl}biphenyl-4-carboxylic acid	ChemBridge	ChemBridge	DMSO
C13	N-{2-[4-(4-cyanophenyl)-3-oxo-1-piperazinyl]-2-oxoethyl}urea	ChemBridge	ChemBridge	DMSO
C14	N-[(5-amino-1H-1,2,4-triazol-3-yl)methyl]-2-(4-methylphenyl)-4-quinolinecarboxamide	ChemBridge	ChemBridge	DMSO

* Compound S12, fosfomycin, is used as a known inhibitor control group. Sigma: Sigma–Aldrich.
